# More Than an Antioxidant: Role of Dietary Astaxanthin on Lipid and Glucose Metabolism in the Liver of Rainbow Trout (*Oncorhynchus mykiss*)

**DOI:** 10.3390/antiox12010136

**Published:** 2023-01-06

**Authors:** Carmen Tatiana Kalinowski, Monica B. Betancor, Silvia Torrecillas, Matthew Sprague, Laurence Larroquet, Vincent Véron, Stéphane Panserat, María Soledad Izquierdo, Sadasivam J. Kaushik, Stéphanie Fontagné-Dicharry

**Affiliations:** 1 Grupo de Investigación en Acuicultura (GIA), Research Institute in Sustainable Aquaculture and Marine Conservation (IU-ECOAQUA), Universidad de Las Palmas de Gran Canaria, 35214 Telde, Spain; 2 Institute of Aquaculture, Faculty of Natural Sciences, University of Stirling, Stirling FK9 4LA, Scotland, UK; 3 INRAE, University of Pau and Pays de l’Adour, NUMEA, 64310 Saint-Pée-sur-Nivelle, France

**Keywords:** astaxanthin, liver, lipid metabolism, glucose metabolism, pentose phosphate pathway, rainbow trout

## Abstract

This study investigated the influence of dietary astaxanthin (AX) on glucose and lipid metabolism in rainbow trout liver. Two iso-nitrogenous and iso-lipidic diets were tested for 12 weeks in rainbow trout with an initial mean weight of 309 g. The S-ASTA diet was supplemented with 100 mg of synthetic AX per kg of feed, whereas the control diet (CTRL) had no AX. Fish fed the S-ASTA diet displayed lower neutral and higher polar lipids in the liver, associated with smaller hepatocytes and lower cytoplasm vacuolization. Dietary AX upregulated adipose triglyceride lipase (*atgl*), hormone-sensitive lipase (*hsl2*) and 1,2-diacylglycerol choline phosphotransferase (*chpt*), and downregulated diacylglycerol acyltransferase (*dgat2*), suggesting the AX’s role in triacylglycerol (TAG) turnover and phospholipid (PL) synthesis. Dietary AX may also affect beta-oxidation with the upregulation of carnitine palmitoyltransferase 1 (*cpt1α2*). Although hepatic cholesterol levels were not affected, dietary AX increased gene expression of sterol regulatory element-binding protein 2 (*srebp2*). Dietary AX upregulated the expression of 6-phosphogluconate dehydrogenase (*6pgdh*) and downregulated pyruvate kinase (*pkl*). Overall, results suggest that dietary AX modulates the oxidative phase of the pentose phosphate pathway and the last step of glycolysis, affecting TAG turnover, β-oxidation, PL and cholesterol synthesis in rainbow trout liver.

## 1. Introduction

Astaxanthin (AX) is a xanthophyll carotenoid used in aquafeeds, predominantly as a pigmenting additive to confer on fish their distinctive and commercially valued skin and fillet colour appearance, directly associated with the final product quality. In salmonids, the essential role of AX has been suggested, especially for critical life stages, but is still controversial [1]. Alongside pigmentation, dietary AX supplementation has other applications as optimum levels and feeding time promote growth and health in several other farmed fish species that do not accumulate high amounts of carotenoids, contrary to salmonids [2,3,4,5]. Among the health benefits in fish, dietary AX improves endogenous enzymatic and non-enzymatic antioxidant responses [6,7], enhances immunological defence mechanisms, and plays an anti-inflammatory role [2,4]. Furthermore, AX can influence fish glucose and lipid metabolism [1,7,8], although the underlying mechanisms remain unclear.

In terrestrial animals, AX has a hypolipidemic effect mitigating symptoms in obesity-related diseases [9,10,11]. Indeed, an in vitro study reported that peroxisome proliferator-activated receptors (PPAR), which regulate lipid and glucose metabolism, are among AX molecular targets [12]. Furthermore, an in vivo study showed that AX activates sterol regulatory element-binding protein 2 (SREBP2), a key regulator of cholesterol metabolism, finding lower plasma cholesterol in animals fed the AX supplemented diet [11]. AX has also been shown to influence sterol regulatory element-binding protein 1c (SREBP1c), a key lipogenesis controlling transcription factor [13,14,15,16]. In addition, dietary AX has a potential antidiabetic role in glucose metabolism, improving insulin sensitivity [17,18,19].

Recent work in Atlantic salmon (*Salmo salar*) revealed that AX’s main effect on hepatic transcriptome was on genes involved in lipid metabolism, specifically on the biosynthesis of terpenoids and steroids [1]. In tiger puffer (*Takifugu rubripes*), dietary AX supplementation upregulated hepatic lipolysis, β-oxidation genes, and downregulated lipogenic related genes [20]. Concerning glucose metabolism, a transcriptome study in Atlantic salmon revealed that dietary AX regulated glucose homeostasis [8]. In Asian seabass (*Lates calcarifer*), dietary AX exerted an anti-hyperglycaemic effect, potentially beneficial in stimulating the insulin sensitivity of fish [4].

Our previous work reported an upregulation of hepatic glucokinase (*gck*) and glucose-6-phosphate dehydrogenase (*g6pdh*), denoting that hepatic glucose and lipid metabolism in rainbow trout (*Oncorhynchus mykiss*) might be sensitive to dietary AX [7]. Indeed, the overexpression of *g6pdh* may reroute glucose flux into the pentose phosphate pathway, thus affecting hepatic glycolysis, as both glucose pathways share the same substrate and run in parallel [21]. Furthermore, the G6PDH and 6-phosphogluconate dehydrogenase (6PGDH) enzymes produce nicotinamide adenine dinucleotide phosphate (NADPH) [22,23]. NADPH plays a fundamental role in antioxidant protection and anabolism with the synthesis of fatty acids, cholesterol, and steroid hormones [24,25,26].

Based on our previous work [7] and the recent literature, we investigated the effect and underlying mechanisms of the action of dietary AX on hepatic glucose and lipid metabolism under normal physiological conditions in fish. Hence, this study is the second part of our previous work [7], focusing on the effect of dietary AX on hepatic glucose and lipid metabolism, independently of the induction of oxidative stress. To get a better insight, the expression of genes involved in glycolysis, oxidative phase of the pentose phosphate pathway (ox-PPP), lipogenesis, lipolysis, and β-oxidation were studied, together with morphology, fatty acid and lipid class composition in the liver tissue.

## 2. Materials and Methods

### 2.1. Experimental Conditions

The experimental design, rearing conditions and diet preparation were previously described [7]. The present study focused on the first 12 weeks of our previous growth trial, where fish were reared under normoxia (8 mg/L) [7]. Briefly, two iso-nitrogenous (41% crude protein), iso-lipidic (23% total lipid) and iso-caloric (24 kJ/g gross energy) diets were formulated, manufactured and tested at the INRAE experimental fish farm in Donzacq (Landes, France, https://doi.org/10.15454/GPYD-AM38, accessed on 3 January 2023) (Table 1). The experimental diets differed in AX content. The CTRL diet had no AX and the S-ASTA diet was supplemented with 100 mg of chemically synthesized AX per kg of feed. The synthetic AX used was Carophyll Pink^®^ containing 10% AX (DSM Nutrition, Village-Neuf, France). All-female diploid rainbow trout with an initial weight of 309 ± 10 g were used and stocked in six 800-L cylindrical fiberglass tanks containing 30 fish each and supplied with flow-through spring water, at 17 °C. Each diet was hand-fed twice a day to visual satiation to triplicate tanks.

### 2.2. Sample Collection

By the end of the feeding trial, ten fish per replicate tank were individually weighed, measured, and sampled [7]. Fish were anesthetized with benzocaine and killed with a blow to the head. Seven fish per tank were dissected to collect viscera with the liver and calculate the viscerosomatic (VSI) and hepatosomatic index (HSI). For gene expression and lipid analysis, half of three liver samples were taken from the seven dissected fish, immediately frozen in liquid nitrogen and stored at −80 °C. The other half of the three liver samples per tank were fixed in buffered formalin for morphological analysis.

### 2.3. Morphological Analysis

Liver samples were dehydrated in a graded ethanol series and embedded in paraffin wax. Paraffin blocks were made and cut (4 μm thick) in a Leica 2055-Autocut microtome (Leica Instruments GmbH, Nussloch, Germany) and stained with hematoxylin and eosin [27]. Micrographs from each slide were taken using a Nikon Microphot-FXA microscope (Nikon Instruments Inc., Melville, NY, USA) incorporated with an Olympus DP50 camera (Olympus Optical Co., LTD, Shinjuku-ku, Tokyo, Japan). The total area of 50 hepatocytes per specimen (450 hepatocytes per experimental diet) was measured as well as the maximum and minimum hepatocyte length, considered the hepatocyte nucleus as a reference point. All the measurements were carried out with ImageJ software (version 1.53e) using arbitrary units.

### 2.4. Total Lipid Content and Fatty Acid Analysis

Hepatic total lipid was extracted from three pooled samples of three livers and measured gravimetrically [28] using dichloromethane instead of chloroform. Fatty acid methyl esters were prepared by acid-catalysed transesterification of total lipid using boron trifluoride in methanol (14%) [29] and analysed in a Varian Chrompack CP-3900 gas chromatograph equipped with a DB Wax fused silica capillary column (30 m × 0·25 mm internal diameter, film thickness 0·25 mm; JW Alltech, France) with helium as the carrier gas (1.4 mL/min). The thermal gradient was 100 to 180 °C at 8 °C/min, 180 to 220 °C at 4 °C/min, and a constant temperature of 220 °C during 20 min. Injector and flame ionization detector temperatures were 260 and 250 °C, respectively. Individual fatty acid methyl esters were identified by comparison with known standards and by reference to published data [30,31]. Data were collected and processed using Chromcard for Windows (version1.19).

### 2.5. Lipid Class Composition

Lipid class composition was determined by high-performance thin-layer chromatography (HPTLC) using 20 × 10 cm plates (VWR, Lutterworth, England). Approximately 1 μg of total lipid was applied on a 3 mm origin and the plates developed in methyl acetate/isopropanol/chloroform/methanol/0.25% aqueous KCl (25:25:25:10:9, by vol.) to half distance. After drying for 20 min, the plate was fully developed with isohexane/diethyl ether/acetic acid (85:15:1, by vol.). Lipid classes were visualized by charring at 160 °C for 15 min after spraying with 3% (*w*/*v*) aqueous cupric acetate containing 8% (*v*/*v*) phosphoric acid and quantified by densitometry using a CAMAG-3 TLC Scanner (version Firmware 1.14.16) [32]. Scanned images were recorded automatically and analysed by computer using winCATS Planar Chromatography Manager (version 1.2.0).

### 2.6. Gene Expression

Total RNA was isolated from the liver using Trizol reagent (Invitrogen, Cergy-Pontoise, France). Quantitative RT-PCR was performed as described previously [33].

Briefly, complementary DNA was generated from 1 mg total RNA using SuperScriptIII RT (Invitrogen) and a mix of oligo (dT)15 and random primers (Promega, Charbonnières, France). Quantitative PCR analyses were performed with 2 µL of the diluted RT reaction mixture (dilution 40) and 4 µL of master mix added with 0.4 mM of each primer (Table 2). Relative quantification of target gene transcripts was performed using elongation factor 1α (*ef1α*) as the reference gene and CTRL as the reference group, using the ΔΔCt method [34].

### 2.7. Statistical Analysis

Data are presented as mean ± standard deviation (SD) or as mean ± standard error of the mean (SEM). Individual fish was the experimental unit for morphometric data and data on gene expression (*n* = 27 with 3 or 9 fish originating from each of the 3 replicate tanks). Tanks (*n* = 3) were used as the experimental unit for data on lipid and fatty acid composition due to the small size of samples, so a pool of 3 fish originating from each of the 3 replicate tanks was used. Differences were considered significant when values of *p* < 0.05. Comparisons between two treatment groups (CTRL and S-ASTA) were assessed by the t-Student test. All statistical analyses were performed using SPSS (IBM, Chicago, IL, USA).

## 3. Results

### 3.1. Liver Weight and Histology

**Figure 1:**
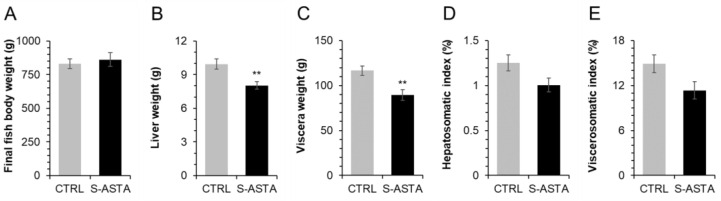
Final body (**A**), liver (**B**) and viscera (**C**) weight with hepatosomatic (**D**) and viscerosomatic (**E**) index of rainbow trout fed a diet without (CTRL) or with astaxanthin (S-ASTA) for 12 weeks. Values are expressed as mean ± SEM (*n* = 27 fish). ** *p* < 0.01 according to *t*-test analysis.

**Figure 2:**
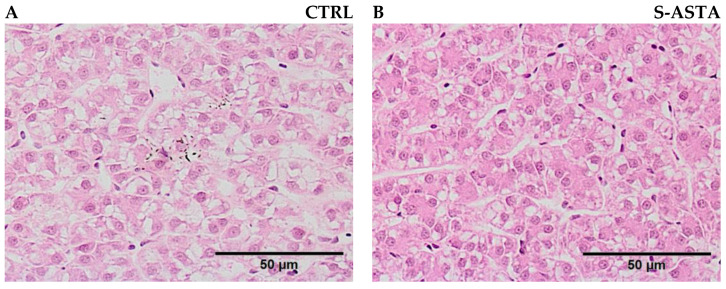
Liver morphology of rainbow trout fed a diet without (CTRL, (**A**)) and with astaxanthin (S-ASTA, (**B**)) for 12 weeks (stained with haematoxylin and eosin).

Fish fed with the S-ASTA diet presented lower liver weight (*p* = 0.002) and viscera weight (*p* = 0.001) than fish fed with the CTRL diet, while final weight was not significantly affected (Figure 1). S-ASTA-fed fish also displayed the lowest HSI and VSI, albeit not significantly (*p* = 0.053 and 0.052, respectively). After a 12-week feeding trial, the liver of rainbow trout fed the S-ASTA diet presented a significantly smaller hepatocyte area, a lower lipid vacuolization, and a more regular-shaped morphology around sinusoidal spaces than the liver of rainbow trout fed the CTRL diet (Figure 2, Table 3).

### 3.2. Liver Lipid Content and Fatty Acid Profile

No significant differences in hepatic total lipid content were observed between the two groups (Table 4). According to the fatty acid composition of the liver total lipid (Table 4), no significant differences in saturated fatty acids (SFA) were noticed between the two groups. A tendency of lower 18:1 (*p* = 0.07), total monounsaturated fatty acids (MUFA) (*p* = 0.09) and ratio between MUFA and SFA (*p* = 0.09) was found in the liver of fish fed S-ASTA. Fish from the S-ASTA group presented significantly lower 18:2 n − 6. With n − 3 polyunsaturated fatty acids (PUFA), only 22:6 n − 3 (docosahexaenoic acid, DHA) showed a significant increase in the S-ASTA diet. A general trend towards more n − 3 PUFA in fish from the S-ASTA group than those from the CTRL treatment resulted in a significantly higher n − 3/n − 6 ratio.

### 3.3. Hepatic Lipid Class Composition

Liver lipid class composition in rainbow trout fed the S-ASTA showed significantly higher polar and lower neutral lipids than fish fed the CTRL diet (Table 5). In hepatic polar lipids, the two main phospholipids (PL), phosphatidylcholine (PC) and phosphatidylethanolamine (PE), were significantly higher in the S-ASTA group than in the CTRL treatment (Table 5). In neutral lipids, triacylglycerols (TAG) and diacylglycerols (DAG) were reduced in fish fed the S-ASTA diet compared to the CTRL group (Table 5).

### 3.4. Hepatic Neutral and Polar Lipid Fatty Acid Profile

Liver neutral and polar lipid fatty acids are presented in Table 6, showing no significant differences in the polar fatty acid profile. However, neutral MUFA were significantly lower in fish from the S-ASTA treatment than in CTRL fish, confirming the tendency observed in total lipid. DHA and 20:5 n − 3 (eicosapentaenoic acid, EPA) from neutral lipids were increased by the S-ASTA diet, as well as the sum of n − 3 PUFA and total PUFA.

### 3.5. Expression of Lipid and Glucose Metabolism Genes in Rainbow Trout Liver

#### 3.5.1. Glucose Metabolism

**Figure 3:**
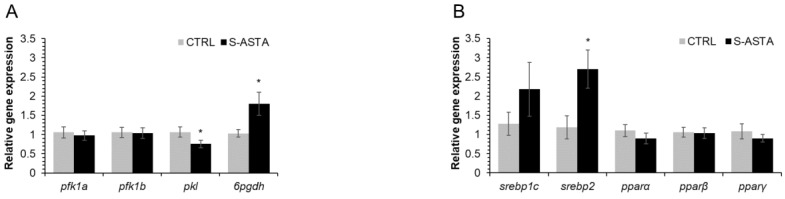

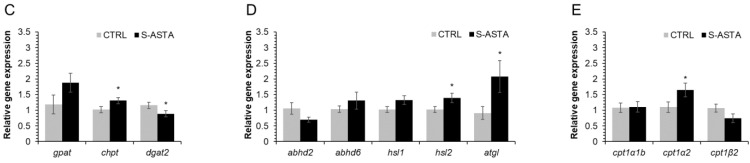
Hepatic expression of genes involved in glycolysis (*pfk1a*, *pfk1b* and *pkl*) and the oxidative phase of the pentose phosphate pathway (*6gdph*, (**A**)), transcription factors regulating lipogenesis (*srebp1c*, *srebp2*, *pparγ*), lipolysis and β-oxidation (*pparα* and *pparβ*, (**B**)), PL and TAG synthesis (**C**), lipolysis (**D**) and β-oxidation (**E**) in rainbow trout fed a diet without (CTRL) or with astaxanthin (S-ASTA) for 12 weeks. Values are expressed as mean ± SEM (*n* = 9 fish). * *p* < 0.05 according to *t*-test analysis.

No significant effect was noticed on phosphofructokinase (*pfk1a*, *pfk1b*) expression levels among treatment groups (Figure 3A). However, pyruvate kinase (*pkl*) showed a significantly lower hepatic expression in rainbow trout fed the S-ASTA diet than fish fed the CTRL diet. On the contrary, *6pgdh* was up-regulated in the S-ASTA livers.

#### 3.5.2. Lipogenesis

Rainbow trout fed the S-ASTA diet presented a significantly higher hepatic expression of *srepbp2*, a transcription factor involved in lipogenesis, than fish fed the CTRL diet (Figure 3B). Furthermore, the hepatic expressions of 1,2-diacylglycerol choline phosphotransferase (*chpt*), an enzyme involved in PC synthesis, and diacylglycerol acyltransferase 2 (*dgat2*), catalyzing the last step in TAG synthesis, were increased and decreased, respectively, by the S-ASTA diet (Figure 3C).

#### 3.5.3. Lipolysis and β-Oxidation

The expressions of *pparα* and *pparβ* were unaffected by dietary treatments (Figure 3B), as were monoacylglycerol lipase (*abhd2* and *abhd6*) mRNA levels (Figure 3D). However, fish from the S-ASTA treatment group had significantly higher hepatic adipose triglyceride lipase (*atgl*) and hormone-sensitive lipase 2 (*hsl2*) expression compared to the levels found in the CTRL group (Figure 3D).

Moreover, hepatic carnitine palmitoyl transferase 1 alpha 2 (*cpt1α2)* expression was significantly increased by dietary AX (Figure 3E).

## 4. Discussion

This study and our previous work [7] provide the first evidence, to our knowledge, of a dietary AX modulating effect on the ox-PPP in rainbow trout liver, upregulating hepatic *g6pdh* [7] and *6pgdh* gene expression. Both ox-PPP dehydrogenases generate NADPH, which maintains the reduced pools of glutathione and thioredoxin [35,36]. Indeed, our previous work reported that rainbow trout fed an AX-supplemented diet presented a higher hepatic ratio between reduced and oxidized glutathione derived from increased *g6pdh* mRNA level and higher glutathione reductase activity and gene expression [7]. Moreover, NADPH is widely assumed to play a role in the reductive biosynthesis of fatty acids, TAG, PL, and cholesterol [24]. Therefore, this study, which is the second part following our previous work [7], aimed to elucidate whether the modulation of dietary AX towards the hepatic ox-PPP (*g6pdh* and *6pgdh*) may also affect lipid metabolism in rainbow trout liver.

Moreover, glucose-6-phosphate (G6P), the ox-PPP substrate, has other metabolic fates in the liver: glycogenesis and glycolysis, obtained by glucokinase, a hepatic enzyme, is also upregulated by dietary AX (with a 3-fold increase of glucokinase b) [7]. Hence, as ox-PPP not only shares the same substrate but also runs parallel with glycolysis [21], dietary AX enhancement of hepatic ox-PPP may have rerouted glucose into the ox-PPP. The inhibition of phosphofructokinase, glyceraldehyde-3-phosphate dehydrogenase and pyruvate kinase denotes the rerouting of glycolysis into the ox-PPP [37]. The altered glycolytic pathway allows cells to divert flux into the ox-PPP to promote NADPH production [37]. Indeed, fish fed on the S-ASTA diet showed lower hepatic mRNA levels of *pkl*. However, fish fed on the S-ASTA diet did not show different mRNA levels of hepatic *pfk1a* and *pfk1b* than CTRL fish. The pyruvate kinase enzyme catalyses pyruvate production, an acetyl CoA precursor. Therefore, the increased flux into the ox-PPP due to dietary AX supplementation may have inhibited the production of hepatic acetyl CoA, a key intermediary for lipid synthesis and ATP production [38]. Moreover, because there is no higher level of hepatic glycogen in this experiment [7], the G6P molecule was mainly transferred to ox-PPP, and glycogenesis was not affected. Based on dietary AX enhancement of hepatic ox-PPP (mRNA levels of *g6pdh* and *6pgdh*), and the possible inhibition of hepatic acetyl CoA production, hepatic lipid metabolism might have been affected.

The transcription factors that regulate lipid homeostasis include SREBP that activate the expression of genes involved in the synthesis and uptake of cholesterol, fatty acids, TAG and PL, as well as the NADPH cofactor required to synthesize these molecules [39]. Among SREBP, SREBP1c activates genes involved in fatty acid and TAG metabolism and induces the expression of genes involved in glucose utilization [40,41]. Studies in terrestrial animals have reported the downregulating effect of AX on *srebp1c* [13,14,16]. However, in a mouse model, dietary AX increased mRNA levels of *srebp1c* in a dose-dependent manner [15]. In this study, the hepatic gene expression of *srebp1c* was higher in the S-ASTA treatment group, although not significant. Among enzymes regulated by *srebp1c* are *g6pdh*, *6pgdh* [40,42], and glucokinase [41]. Therefore, the higher mRNA levels of *srepb1c* found in fish fed S-ASTA diet could have influenced hepatic mRNA levels of glucokinase and *g6pdh* reported in our previous work [7] and *6pgdh* found in this study. From the molecular perspective, it seems that dietary AX influences hepatic lipogenesis. However, biochemical results do not corroborate molecular findings as no differences in hepatic lipid content or fatty acid synthesis products such as SFA and MUFA in total lipids and lipid classes were found among dietary treatment groups. Nevertheless, fish fed the S-ASTA diet showed a higher hepatic content of DHA. Carotenoids seem to increase the biosynthesis of n − 3 and n − 6 PUFA [43], although, in this study, the S-ASTA diet did not affect the levels of any of the precursors of this fatty acid. As n − 3 long-chain PUFA are readily oxidized, the higher DHA levels found in the liver of S-ASTA fed fish could be due to the antioxidant activity of AX. Indeed, liver thiobarbituric acid-reactive substance (TBARS) values were reduced by 15% due to AX supplementation [7]. Another significant finding in rainbow trout fed the S-ASTA diet was the higher n − 3/n − 6 ratio. An adequate balance of n − 3 and n − 6 PUFA is fundamental for several physiological functions promoting a better liver status in rainbow trout, as both fatty acid groups are substrates for different enzymes involved in fish lipid metabolism [44]. As CTRL and S-ASTA diets were formulated with the same batch of ingredients, including fish oil, similar dietary fatty acid profiles can be expected, suggesting that our results on liver could be due to dietary AX supplementation. Therefore, neither dietary AX enhancement of the hepatic ox-PPP nor the glycolytic rerouting seems to affect hepatic fatty acid synthesis in rainbow trout liver. The use of NADPH on anabolic reactions or the maintenance of cellular redox homeostasis depends on the cell requirements [45]. In the present study, we previously suggested that hepatic ox-PPP enhancement improved oxidative stress protection by increasing glutathione reductase activity and gene expression, leading to higher ratio between reduced and oxidized glutathione (GSH/GSSG) and lower TBARS values [7].

Regarding hepatic glycerolipid synthesis, fish fed on the S-ASTA diet showed lower hepatic mRNA levels of *dgat2*, the rate-limiting enzyme for TAG formation, which modulates the last step in TAG synthesis. The reaction catalysed by DGAT is crucial as it is a branching point for hydrocarbon flow towards either the TAG pathway or PL pathway [46]. Therefore, this finding suggests that dietary AX inhibits hepatic TAG synthesis and enhances hepatic PL synthesis. Indeed, increasing hepatic mRNA levels of *chpt* involved in the synthesis of PC from DAG were found in rainbow trout fed the S-ASTA diet, as DAG can act as a precursor of PC and PE [47]. Similarly, in tiger puffer, AX supplementation downregulated the gene expression of *dgat1* [20]. Nevertheless, in broiler chicken, dietary AX upregulated the *dgat2* mRNA [16]. Hepatic lipid class composition corroborates our molecular findings, reporting that rainbow trout fed on the S-ASTA diet presented higher hepatic PL (PC and PE) and lower hepatic total neutral lipids than fish from the CTRL treatment. In mice models, the perturbation of pyruvate kinase affects lipogenesis, lowering liver TAG and cholesterol [48]. Therefore, dietary AX downregulation of hepatic *pkl* may have caused the lowering of hepatic TAG synthesis in rainbow trout liver. Furthermore, overall molecular TAG metabolism results denote the role of dietary AX in modulating TAG turnover in rainbow trout liver. Consequently, this affects hepatic PL composition since TAG turnover seems essential in maintaining the pool size of PL [49].

Concerning SREBP2, fish fed the S-ASTA diet presented the highest mRNA levels of hepatic *srebp2*, an activator of cholesterol biosynthetic enzymes [39]. In agreement with our results, AX supplementation increased *srebp2* mRNA levels in mice [11]. The increase of hepatic mRNA levels of *srebp2* observed in fish fed the S-ASTA diet could be a compensatory response linked to the downregulating effect of dietary AX supplementation on hepatic *pkl*, lowering hepatic acetyl CoA and affecting cholesterol biosynthesis. Moreover, the rate-limiting enzyme of cholesterol biosynthesis, 3-hydroxy-3-methylglutaryl CoA reductase (not evaluated in this study), requires NADPH as a cofactor. Therefore, the increase in hepatic NADPH produced by AX enhancement of the hepatic ox-PPP might have also affected the activity of this cholesterol-synthesizing enzyme. However, at the end of the trial, cholesterol concentrations in the liver of rainbow trout were not significantly different among dietary treatments. In a study with Atlantic salmon fed diets with low content of marine ingredients, only differing in AX concentrations (48 and <1 mg/kg feed), fish fed the diet with low AX levels showed higher biosynthesis of hepatic steroids [1]. In diets with low fishmeal and fish oil, there is low cholesterol content, and there is a need for endogenous synthesis of cholesterol [50,51,52]. Therefore, results found in Atlantic salmon liver [1] reinforce the dietary AX role in cholesterol homeostasis.

Furthermore, several studies have suggested the implication of dietary AX in fatty acid utilization via the activation of *cpt1* and associated with enhanced lipolysis and sparing of glycogen [10,53,54]. Indeed, this study found an effect of AX supplementation on hepatic lipolysis and β-oxidation. Rainbow trout fed the S-ASTA diet showed higher mRNA levels of hepatic *atgl*, *hsl1* and *hsl2*. Indeed, rainbow trout fed the S-ASTA showed lower hepatic TAG. Both enzymes are involved in lipolysis, mobilizing TAG and under the control of the PPARγ transcription factor [55,56]. However, in the present study, hepatic *pparγ* was not affected by the S-ASTA diet. Most studies have reported dietary AX as an antagonist of PPARγ [11,12]. The increased hydrolytic cleavage of hepatic TAG due to dietary AX supplementation increased the availability of fatty acids, promoting β-oxidation, resulting in fish fed on the S-ASTA diet presenting higher mRNA levels of *cpt1α2*. In fact, rainbow trout fed the S-ASTA diet showed lower hepatic MUFA in the neutral fraction than fish fed the CTRL diet, which are among the main substrates for mitochondrial β-oxidation in fish [57]. However, dietary AX did not affect hepatic *pparα*, a transcription factor stimulating fatty acid catabolism [58]. Similarly, in tiger puffer, dietary AX increased the hepatic mRNA expression of genes involved in β-oxidation and monoacylglycerol hydrolysis [20]. In mice, dietary AX also upregulated the expression of *cpt1*, suggesting that AX may activate *pparα* [11,59]. The enhancement of hepatic lipolysis and β-oxidation found in this work suggests that dietary AX may stimulate the use of fatty acids for ATP generation, to compensate for the lowering of ATP production derived from the rerouting of hepatic glucose toward the pentose phosphate pathway to maintain hepatic energy homeostasis.

Biological and histological results are also in agreement with molecular observations, finding that liver and visceral weights were lower in fish fed the S-ASTA diet. In Atlantic salmon fed diets with low content of marine ingredients, dietary AX decreased HSI but not the visceral fat score [1]. Moreover, mice fed a high-fat diet supplemented with AX displayed reduced body weight, adipose tissue weight, liver weight, and liver TAG [10,54]. Furthermore, liver histology strengthens our results, as fish fed the S-ASTA diet presented lower hepatocyte area and lower nuclear displacement than rainbow trout fed the CTRL diet. Indeed, studies on diverse fish species have also reported a positive influence of AX on liver histology. Dietary AX has been shown to improve liver structure and metabolism in *Oreochromis niloticus* and *Colisa labiosa* [60]. In Eurasian perch (*Perca fluviatilis* L.), AX partially abrogated the effects of a high-fat diet [61].

## 5. Conclusions

This study is the first to document the modulating effect of dietary AX on the ox-PPP and the last step of the glycolytic pathway in rainbow trout liver. Both findings influenced TAG turnover, β-oxidation, PL, and cholesterol synthesis in rainbow trout liver. Overall, dietary AX positively influenced rainbow trout liver physiology and is a suitable feed additive to help address aquaculture challenges related to hepatic health, as a fatty liver is caused by an imbalance between lipogenesis and lipolysis, and oxidative stress. However, further studies testing different AX levels on salmonids and other fish species are worth evaluating, to define the minimal efficient level as AX represents a significant fraction of the feed cost. The sustainability of the aquaculture industry, indeed, requires realistic functional ingredients.

## Figures and Tables

**Table 1:** Formulation of the experimental diets.

Ingredients (%)	CTRL	S-ASTA
Fishmeal	23	23
Plant meals ^1^	51.8	51.8
Fish oil	19	19
Soybean lecithin	2	2
Vitamin premix ^2^	1	1
Mineral premix ^3^	3	3
Cellulose	0.2	0.1
Carophyll Pink 10% DSM	-	0.1

^1^ Plant meals (% diet): wheat gluten meal, 20; soybean protein concentrate, 20; rapeseed meal, 10; whole wheat, 11.8. ^2^ Vitamin premix (per kg diet): retinyl acetate, 5000 IU; cholecalciferol, 2500 IU; DL α-tocopheryl acetate, 50 IU; sodium menadione bisulfate, 10 mg; thiamin-HCl, 1 mg; ribo-flavin, 4 mg; niacin, 10 mg; D-calcium pantothenate, 20 mg; pyridoxine-HCl, 3 mg; D-biotin, 0.2 mg; folic acid, 1 mg; cyanocobalamin, 0.01 mg; L-ascorbyl-2-polyphosphate, 50 mg; myo-inositol, 300 mg; choline, 1000 mg. All ingredients were diluted with α-cellulose. ^3^ Mineral premix (per kg diet): CaHPO_4_·2H_2_O, 25 g; CaCO_3_, 2.15 g; MgO, 1.24 g; KCl, 0.9 g; NaCl, 0.4 g; FeSO_4_·7H_2_O, 0.2 g; ZnSO_4_·7H_2_O, 40 mg; MnSO_4_·H_2_O, 30 mg; CuSO_4_·5H_2_O, 30 mg; NaF, 10 mg; KI, 0.4 mg; Na_2_SeO_3_, 0.3 mg; CoCl_2_·6H_2_O, 0.2 mg.

**Table 2:** PCR primers used to assay gene expression by real-time quantitative polymerase chain reaction.

Gene	Forward Primer Sequence	Reverse Primer Sequence	Amplicon Size	Accession Number
*ef1α*	tcctctggtcgtttcgctg	acccgagggacatcctgtg	159	AF498320.1
*pfkla*	gatccctgccaccatcagta	gtaaccacagtagcctccca	166	XM_036959537.1
*pfklb*	agtgctcgctgtaaggtctt	gtgatccggcctttctgaac	182	XM_036959534.1
*pkl*	ccatcgtcgcggtaacaaga	gcccctggcctttcctatgt	158	XM_036968223.1
*6pgdh*	atgccagggggacacaaaga	caaaagcctgtgccatcacg	238	XM_021616114.2
*srebp1c*	catgcgcaggttgtttctt	gatgtgttcgtgtgggactg	74	XM_021624594.1
*srebp2*	taggccccaaagggataag	tcagacacgacgagcacaa	179	XM_021558051.2
*pparα*	ctggagctggatgacagtga	ggcaagtttttgcagcagat	192	AY494835.1
*pparβ*	ctggagctggatgacagtga	gtcagccatcttgttgagca	195	AY356399.1
*pparγ*	cccacggaaactcaccgttt	ggatctggatacggcggaag	168	CA345564.1
*gpat*	tgccacacggtacctattga	ccacaggggtgagtttgagt	168	XM_021565307.2 ^1^
*chpt*	ggccaagatcaccaacaaat	aaagacaggatcagcgcaat	162	CA355941.1
*dgat2*	ggaacacccccaaacaaggt	agatcccatgggggtggtag	156	LOC110533663
*abhd2*	ccacctttgacctcttcgag	gcttctcactgtggttacca	96	XM_021565941.2 ^2^
*abdh6*	tccctatcctggccttcttt	ccggtagcctctgttctcag	125	XM_036984302.1 ^3^
*hsl1*	gtcctagggtcatggtcatcgt	tctctggtgggccttgttgt	65	HQ225622.1
*hsl2*	catcgtcaagaacccgtttg	gcggtagtcctctcagtaggtcat	60	HQ225623.1
*atgl*	cgtgtccgagttcaagtc	ggagagatgctgatggtg	174	BX318925
*cpt1α1b*	cgcttcaagaatggggtgat	caaccacctgctgtttctca	187	AJ619768.1
*cpt1α2*	ccgttcctaacagaggtgct	acactccgtagccatcgtct	154	AJ620356.1
*cpt1β2*	gccgcaaactagagagagga	cccgtagtacagccacacct	199	AF327058.3

*ef1*α, elongation factor 1α; *pfk1a* and *pfk1b*, 6-phosphofructo-1-kinase a et b; *pkl*, pyruvate kinase; *6pgdh*, 6-phosphogluconate dehydrogenase; *srebp1c* and *srebp2*, sterol regulatory element-binding 1c and 2; *pparα*, *pparβ* and *pparγ*, peroxisome proliferator-activated receptor α, β and γ; *gpat*, glycero-3-phosphate acyltransferase, mitochondrial; *chpt*, 1,2-diacylglycerol choline phosphotransferase; *dgat2*, diacylglycerol acyltransferase 2; *abhd2* and *abhd6*, monoacylglycerol lipase (abhydrolase domain containing 2, acylglycerol lipase and abhydrolase domain containing 6, acylglycerol lipase); *hsl1* and *hsl2*, hormone sensitive lipase 1 and 2; *atgl*, adipose triglyceride lipase; *cpt1α1b*, *cpt1α2* and *cpt1β2*, carnitine palmitoyl transferase 1 alpha 1b, alpha 2 and beta 2. ^1^ Additionally, also XM_036946572.1. ^2^ Additionally, also XM_021565950.2 and XM_021565922.2. ^3^ Additionally, also XM_036984303.1.

**Table 3:** Hepatocyte morphometry of rainbow trout fed a diet without (CTRL) and with astaxanthin (S-ASTA) for 12 weeks.

Dietary Groups	CTRL	S-ASTA
Hepatocyte area	1878 ± 24	1705 ± 20 *
Minimum length	38.9 ± 0.3	39.0 ± 0.3
Maximum length	53.8 ± 0.4	51.5 ± 0.4 *
Difference length	14.9 ± 0.4	12.5 ± 0.4 *

Results are expressed as means ± SD (*n* = 9 fish; arbitrary units). * *p* < 0.05 according to *t*-test analysis.

**Table 4:** Liver lipid content and total lipid fatty acid composition (% of total fatty acids) of rainbow trout fed a diet without (CTRL) and with astaxanthin (S-ASTA) for 12 weeks.

Dietary Groups	CTRL	S-ASTA
Liver lipid content (%)	5.9 ± 0.5	5.1 ± 0.4
Liver fatty acids (% of total fatty acids)		
14:0	1.5 ± 0.2	1.4 ± 0.2
16:0	13.7 ± 0.9	14.3 ± 0.3
18:0	5.2 ± 1.8	6.3 ± 1.2
SFA ^1^	21.1 ± 0.8	22.9 ± 1.2
16:1	4.8 ± 1.1	3.6 ± 1.1
18:1	21.2 ± 1.8	17.7 ± 1.8
20:1	3.6 ± 0.2	3.1 ± 0.3 *
MUFA ^2^	30.2 ± 2.7	24.9 ± 3.3
MUFA/SFA ratio	1.4 ± 0.2	1.0 ± 0.2
18:2 n − 6	3.9 ± 0.1	3.6 ± 0.2 *
20:2 n − 6	1.5 ± 0.1	1.4 ± 0.1
20:4 n − 6	4.0 ± 0.3	4.6 ± 0.6
22:5 n − 6	0.9 ± 0.1	1.0 ± 0.0 *
n − 6 PUFA ^3^	11.4 ± 0.4	11.6 ± 0.7
18:3 n − 3	0.5 ± 0.0	0.5 ± 0.1
20:4 n − 3	0.4 ± 0.0	0.4 ± 0.0
20:5 n − 3	4.3 ± 1.0	4.8 ± 0.7
22:5 n − 3	2.4 ± 0.5	2.3 ± 0.1
22:6 n − 3	26.6 ± 1.2	29.5 ± 1.3 *
EPA + DHA	30.9 ± 1.7	34.3 ± 1.9
n−3 PUFA ^4^	34.9 ± 1.9	38.0 ± 1.9
PUFA ^5^	47.5 ± 2.2	51.0 ± 2.4
N − 3/n − 6 ratio	3.0 ± 0.1	3.3 ± 0.1 *

Results are expressed as means ± SD (*n* = 3 pools of 3 fish originating from each of the 3 replicate tanks). A SD of 0.0 implies a SD < 0.05. * *p* < 0.05 according to *t*-test analysis. SFA, saturated fatty acids; MUFA, monounsaturated fatty acids; PUFA, polyunsaturated fatty acids; DHA, docosahexaenoic acid (22:6 n − 3); EPA, eicosapentaenoic acid (20:5 n − 3). ^1^ Total includes 15:0, 17:0 and 20:0. ^2^ Total includes 17:1 and 22:1. ^3^ Total includes 18:3 n − 6, 20:3 n − 6, 22:2 n − 6 and 22:4 n − 6. ^4^ Total includes 18:4 n − 3, 20:3 n − 3 and 21:5n − 3. ^5^ Total includes n − 6 PUFA, n − 3 PUFA, 16:2n − 4, 16:3n − 4, 18:2n − 4 and 18:3n − 4.

**Table 5:** Liver lipid class composition (% of total lipid) of rainbow trout fed a diet without (CTRL) and with astaxanthin (S-ASTA) for 12 weeks.

Dietary Groups	CTRL	S-ASTA
Phosphatidylcholine	19.0 ± 0.3	25.0 ± 3.2 *
Phosphatidylethanolamine	8.8 ± 1.5	11.6 ± 1.5 *
Phosphatidylinositol	4.1 ± 0.5	5.2 ± 1.0
Phosphatidylserine	1.7 ± 0.5	2.1 ± 0.2
Phosphatidic acid and cardiolipin	2.8 ± 0.2	3.4 ± 0.5
Sphingomyelin	1.1 ± 0.1	1.3 ± 0.2
Lysophosphatidylcholine	0.0 ± 0.0	0.0 ± 0.0
Unknown polar lipids	1.0 ± 0.2	1.2 ± 0.5
Total polar lipids	38.9 ± 1.2	51.0 ± 7.2 *
Triacylglycerols	33.4 ± 4.5	24.9 ± 6.2
Cholesterol and sterols	14.0 ± 0.5	15.3 ± 1.4
Wax and sterol esters	9.2 ± 3.6	6.0 ± 3.2
Diacylglycerols	3.5 ± 0.3	2.6 ± 0.8
Free fatty acids	1.1 ± 1.3	0.2 ± 0.3
Total neutral lipids	61.1 ± 1.2	49.0 ± 7.2 *
Triacylglycerol/cholesterol ratio	2.4 ± 0.2	1.7 ± 0.5

Results are expressed as means ± SD (*n* = 3 pools of 3 fish originating from each of the 3 replicate tanks). * *p* < 0.05 according to *t*-test analysis.

**Table 6:** Liver neutral and polar lipid fatty acid composition (% of total fatty acids) of rainbow trout fed a diet without (CTRL) and with astaxanthin (S-ASTA) for 12 weeks.

	Neutral Lipids		Polar Lipids	
Dietary Groups	CTRL	S-ASTA	CTRL	S-ASTA
14:0	2.3 ± 0.1	2.2 ± 0.3	1.0 ± 0.2	0.9 ± 0.1
16:0	13.1 ± 0.6	12.7 ± 0.6	15.5 ± 1.4	15.8 ± 0.6
18:0	2.2 ± 0.8	2.4 ± 0.6	8.5 ± 2.5	9.2 ± 1.4
SFA ^1^	18.4 ± 0.1	18.3 ± 0.3	26.0 ± 1.0	26.9 ± 1.6
16:1 n − 7	8.7 ± 1.7	7.0 ± 1.4	1.7 ± 0.3	1.6 ± 0.3
18:1 n − 9	28.5 ± 1.7	27.2 ± 1.2	9.7 ± 1.3	9.6 ± 1.1
18:1 n − 7	5.7 ± 1.0	5.2 ± 0.5	3.8 ± 0.7	3.2 ± 0.6
20:1 n − 9	3.5 ± 0.2	3.5 ± 0.2	3.3 ± 0.3	2.6 ± 0.4
MUFA ^2^	48.8 ± 1.3	45.4 ± 1.5 *	20.0 ± 1.8	18.5 ± 1.8
MUFA/SFA ratio	2.7 ± 0.1	2.4 ± 0.1	0.8 ± 0.1	0.7 ± 0.1
18:2 n − 6	5.9 ± 0.2	6.1 ± 0.1	2.8 ± 0.1	2.8 ± 0.2
20:2 n − 6	1.2 ± 0.1	1.3 ± 0.1	1.8 ± 0.1	1.5 ± 0.2
20:4 n − 6	1.3 ± 0.1	1.5 ± 0.2	6.3 ± 0.2	6.2 ± 0.2
22:5 n − 6	0.6 ± 0.0	0.7 ± 0.0 *	1.2 ± 0.0	1.2 ± 0.1
n − 6 PUFA ^3^	9.8 ± 0.4	10.4 ± 1.4	12.7 ± 0.1	12.4 ± 0.4
18:3 n − 3	0.9 ± 0.1	1.0 ± 0.3	0.3 ± 0.0	0.3 ± 0.1
20:4 n − 3	0.5 ± 0.1	0.7 ± 0.2	0.3 ± 0.0	0.3 ± 0.0
20:5 n − 3	2.8 ± 0.6	3.2 ± 0.4	5.5 ± 1.1	5.5 ± 0.5
22:5 n − 3	3.2 ± 0.7	3.7 ± 0.4	1.6 ± 0.3	1.7 ± 0.1
22:6 n − 3	14.7 ± 0.6	16.4 ± 1.1	33.3 ± 0.9	34.1 ± 0.3
EPA + DHA	17.5 ± 0.3	19.5 ± 0.7 **	38.8 ± 0.4	39.6 ± 0.7
n − 3 PUFA ^4^	22.9 ± 0.9	26.0 ± 0.3 **	41.3 ± 0.7	42.2 ± 0.8
PUFA ^5^	32.8 ± 1.2	36.2 ± 1.5 *	54.0 ± 0.8	54.6 ± 0.9
n − 3/n − 6 ratio	2.3 ± 0.0	2.5 ± 0.3	3.2 ± 0.0	3.4 ± 0.1

Results are expressed as means ± SD (*n* = 3 pools of 3 fish originating from each of the 3 replicate tanks). A SD of 0.0 implies a SD < 0.05. * *p* < 0.05; ** *p* < 0.01 according to *t*-test analysis within each lipid class (neutral or polar lipids). SFA, saturated fatty acids; MUFA, monounsaturated fatty acids; PUFA, polyunsaturated fatty acids; DHA, docosahexaenoic acid (22:6 n − 3); EPA, eicosapentaenoic acid (20:5 n − 3). ^1^ Total includes 15:0, 17:0 and 20:0. ^2^ Total includes 16:1 n − 9, 17:1, 20:1 n − 11, 22:1 n − 11 and 24:1 n − 9. ^3^ Total includes 18:3 n − 6, 20:3 n − 6 and 22:4 n − 6. ^4^ Total includes 18:4 n − 3, 20:3 n − 3 and 21:5n − 3. ^5^ Total includes n − 6 and n − 3 PUFA.

## Data Availability

Data are available upon request to the corresponding author.
